# Supplementary material for: Effects of Spiro-Cyclohexane Substitution of Nitroxyl Biradicals on Dynamic Nuclear Polarization
Source: Molecules. 2022 May 19;27(10):3252. doi: 10.3390/molecules27103252 (PMC9143461; doi:10.3390/molecules27103252)
Supplement: Supplementary file 1 [file molecules-27-03252-s001.zip › molecules-1734503-supplementary.pdf]

Supporting Information

Effects of Spiro-Cyclohexane Substitution of Nitroxyl Biradicals on Dynamic Nuclear Polarization

Nargiz B. Asanbaeva[a], Larisa Yu. Gurskaya[a], Yuliya F. Polienko[a], Tatyana V. Rybalova[a], Maxim S. Kazantsev[a], Alexey A. Dmitriev[b], Nina P. Gritsan[b], Nadia Haro-Mares[c], Torsten Gutmann[c], Gerd Buntkowsky[c], Evgeny V. Tretyakov[d], Elena G. Bagryanskaya[a*]

[a]*N.N. Vorozhtsov Institute of Organic Chemistry, 9 Ac. Lavrentiev Avenue, Novosibirsk 630090, Russian Federation*

[b]*V.V. Voevodsky Institute of Chemical Kinetics and Combustion, Siberian Branch of the Russian Academy of Sciences, 3 Institutskaya Str, 630090 Novosibirsk, Russian Federation*

[c]*TU Darmstadt; Eduard-Zintl-Institute for Inorganic and Physical Chemistry, Alarich-Weiss-Straße 8, 64287 Darmstadt, Germany*

[d]*N.D. Zelinsky Institute of Organic Chemistry, Russian Academy of Sciences, 47 Leninsky prosp., 119991 Moscow, Russian Federation*

X-ray crystallography

XRD data were obtained at room temperature on a Bruker Kappa Apex II CCD diffractometer with Mo Kα radiation (λ = 0.71073 Å) and a graphite monochromator. Absorption corrections were applied empirically using *SADABS* programs [1]. The structures were solved by direct methods using the *SHELX-97* [2] programs set and refined by full-matrix least-squares method against all F^2 in anisotropic (beside the atoms H) approximation using the *SHELXL2014*/7 programs set [3]. The H atoms positions were calculated with the riding model. The asymmetric unit contains also molecule of chloroform. The obtained crystal structure geometry and short contacts between non-bonded atoms were analyzed using the PLATON program [4].

Crystal solvate of diradical **4** is triclinic, space group P-1, *a* 12.4262(5), *b* 13.3902(5), *c* 13.7759(6) Å, *α* 67.121(2), *β* 67.676(2), *γ* 77.066(2)º, *V* 1945.7(1) Å^3, Z 2, $C_{41}H_{51}Cl_3N_6O_2Fe$, Dc 1.333 g/cm^3, μ 0.638 mm^{-1}, F(000) 864, crystal size 0.20 × 0.06 × 0.05 mm^3, independent reflections 8868, R 0.0973,R2 0.2002 for, S 1.028 for all reflections (R 0.0657, wR2 0.1767 for 6296 I>2σ), largest difference peak and hole 1.769 and -1.412 e.Å^{-3}.

CCDC 2168987 contains the supplementary crystallographic data for this paper. These data can be obtained free of charge *via* http://www.ccdc.cam.ac.uk/cgi-bin/catreq.cgi, or from the Cambridge Crystallographic Data Centre, 12 Union Road, Cambridge CB2 1EZ, UK; fax: (+44) 1223 336 033; or e-mail: deposit@ccdc.cam.ac.uk

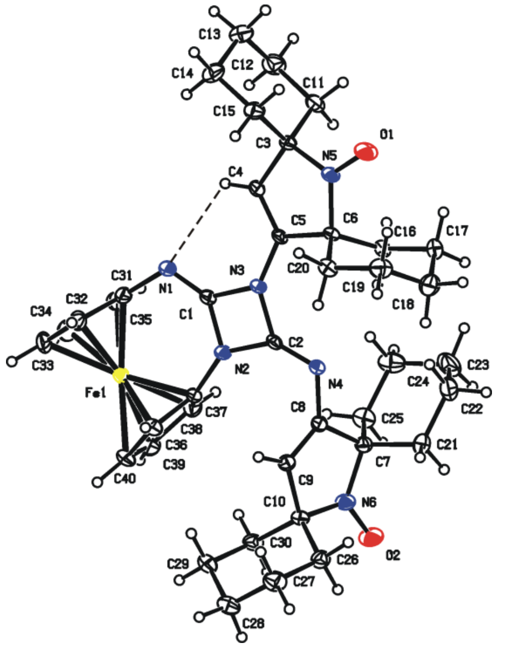

Figure S1. Molecular structure of diradical **4** with the atom-labelling scheme. Displacement ellipsoids are drawn at the 30% probability level.

Intermolecular short contact Cl3…O2 equaling to 3.067(3) Å is observed in solvate of **4** (sum of VDW radii [5] is 3.34 Å) (Figure S2). Solvates are bound through H-bond C1S-H...N1 (Table 3) between chloroform and 2,4-diimino-1,3-diazetidine moiety into chains directed along the (*b*-*c*). The chains, in turn, are formally bound *via* C40-H...O1 h-bond in (*b*, *c*) plain and then *via* C32-H...O1 and C38-H...O2 h-bonds along axis *a* into 3D-structure.

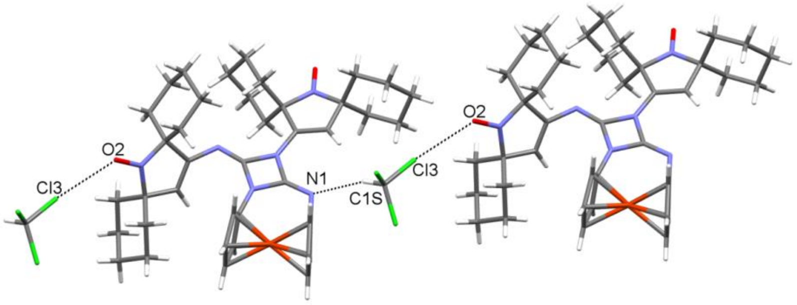

Figure S2. Fragment of molecular chain directed along (*b*-*c*).

Table S1. Intermolecular hydrogen bonds in crystals of diradical **4** solvate.

	H…O, Å	C…O, Å	C-H…O, º
C1S-H...N1	2.59	3.536(6)	161
C32-H...O1	2.59	3.485(6)	163
C40-H...O1	2.51	3.274(5)	140
C38-H...O2	2.61	3.403(5)	144

References

1. Sheldrick, G.M. *SADABS, Program for Empirical Absorption Correction of Area Detector Data*; 1996;
2. Sheldrick, G.M. A Short History of {\it SHELX}. *Acta Crystallogr. Sect. A* **2008**, *64*, 112–122, doi:10.1107/S0108767307043930.
3. Sheldrick, G.M. Crystal Structure Refinement with SHELXL. *Acta Crystallogr. Sect. C Struct. Chem.* **2015**, *71*, 3–8, doi:10.1107/S2053229614024218.
4. Spek, A. Structure Validation in Chemical Crystallography. *Acta Crystallogr. D. Biol. Crystallogr.* **2009**, *65*, 148–155, doi:10.1107/S090744490804362X.
5. Rowland, R.S.; Taylor, R. Intermolecular Nonbonded Contact Distances in Organic Crystal Structures: Comparison with Distances Expected from van Der Waals Radii. *J. Phys. Chem.* **1996**, *100*, 7384–7391, doi:10.1021/jp953141+.
